# Complete genome sequence of a serotype 11A, ST62 *Streptococcus pneumoniae *invasive isolate

**DOI:** 10.1186/1471-2180-11-25

**Published:** 2011-02-01

**Authors:** Romina Camilli, Raoul JP Bonnal, Maria Del Grosso, Michele Iacono, Giorgio Corti, Ermanno Rizzi, Magda Marchetti, Laura Mulas, Francesco Iannelli, Fabiana Superti, Marco R Oggioni, Gianluca De Bellis, Annalisa Pantosti

**Affiliations:** 1 Department of Infectious, Parasitic and Immune-mediated Diseases, Istituto Superiore di Sanità, Rome, Italy; 2 Italian National Research Council, Institute for Biomedical Technologies, Milan, Italy; 3 Department of Technology and Health, Istituto Superiore di Sanità, Rome, Italy; 4 Department of Molecular Biology, University of Siena, Siena, Italy

## Abstract

**Background:**

*Streptococcus pneumoniae *is an important human pathogen representing a major cause of morbidity and mortality worldwide. We sequenced the genome of a serotype 11A, ST62 *S. pneumoniae *invasive isolate (AP200), that was erythromycin-resistant due to the presence of the *erm*(TR) determinant, and carried out analysis of the genome organization and comparison with other pneumococcal genomes.

**Results:**

The genome sequence of *S. pneumoniae *AP200 is 2,130,580 base pair in length. The genome carries 2216 coding sequences (CDS), 56 tRNA, and 12 rRNA genes. Of the CDSs, 72.9% have a predicted biological known function. AP200 contains the pilus islet 2 and, although its phenotype corresponds to serotype 11A, it contains an 11D capsular locus. Chromosomal rearrangements resulting from a large inversion across the replication axis, and horizontal gene transfer events were observed. The chromosomal inversion is likely implicated in the rebalance of the chromosomal architecture affected by the insertions of two large exogenous elements, the *erm*(TR)-carrying Tn*1806 *and a functional prophage designated ϕSpn_200. Tn*1806 *is 52,457 bp in size and comprises 49 ORFs. Comparative analysis of Tn*1806 *revealed the presence of a similar genetic element or part of it in related species such as *Streptococcus pyogenes *and also in the anaerobic species *Finegoldia magna, Anaerococcus prevotii *and *Clostridium difficile*. The genome of ϕSpn_200 is 35,989 bp in size and is organized in 47 ORFs grouped into five functional modules. Prophages similar to ϕSpn_200 were found in pneumococci and in other streptococcal species, showing a high degree of exchange of functional modules. ϕSpn_200 viral particles have morphologic characteristics typical of the *Siphoviridae *family and are capable of infecting a pneumococcal recipient strain.

**Conclusions:**

The sequence of *S. pneumoniae *AP200 chromosome revealed a dynamic genome, characterized by chromosomal rearrangements and horizontal gene transfers. The overall diversity of AP200 is driven mainly by the presence of the exogenous elements Tn*1806 *and ϕSpn_200 that show large gene exchanges with other genetic elements of different bacterial species. These genetic elements likely provide AP200 with additional genes, such as those conferring antibiotic-resistance, promoting its adaptation to the environment.

## Background

*Streptococcus pneumoniae *is a Gram-positive human pathogen responsible for serious diseases such as pneumonia, meningitis and sepsis [1]. The reservoir of *S. pneumoniae *is represented by asymptomatic carriage in the nasopharynx, particularly in young children [2]. The mechanism by which pneumococci become pathogenic is poorly understood, and probably depends on a complex interaction between bacterial virulence factors [3] and the patients' immunological response [4]. The emergence of antibiotic-resistant *S. pneumoniae *strains has represented an additional problem in the management of pneumococcal infections [5]. *S. pneumoniae *strains that are resistant to commonly used antibiotics such as penicillins and macrolides are isolated from all areas of the globe [6].

So far, more than 90 different *S. pneumoniae *serotypes have been recognized on the basis of immunochemical differences in the polysaccharide capsule and their number is probably due to increase [7-10].

After implementation of the 7-valent pneumococcal conjugate vaccine (PCV7) in the USA, a profound change in the distribution of the serotypes colonizing children [11] and causing diseases has been observed [12,13]. Some of the so-called non-vaccine serotypes, that is serotypes not included in the pneumococcal conjugate vaccine, are becoming increasingly common [13] and increasingly antibiotic resistant [14,15].

Novel insights into the genome organization and metabolism of *S. pneumoniae *have been gained from analysis of complete genomes. To date, 23 pneumococcal strains, belonging to different serotypes including 1, 2, 3, 4, 5, 6B, 14, 19A, 19F and 23F, have been completely sequenced, while other strains have been partially sequenced or are currently under way http://genome.microbio.uab.edu/strep/info/; http://www.sanger.ac.uk/Projects/S_pneumoniae/;http://cmr.tigr.org; http://www.genomesonline.org http://www.ncbi.nlm.nih.gov/genome/.

We have sequenced the complete genome of a clinical isolate (AP200) belonging to serotype 11A, Sequence Type (ST) 62, a non-vaccine serotype that is currently on the rise, being one of the most prevalent serotypes isolated both from carriage [16,17] and invasive diseases [18] in North America following the introduction of PCV7. According to Brueggemann *et al*. [19], serotype 11A is more associated with asymptomatic carriage than with invasive disease indicating a relatively low disease potential. However, serotype 11A strains, especially those belonging to ST62, are able to cause invasive disease with significant mortality [19,20]. The draft genomes of two other serotype 11A, ST62 pneumococcal strains, SP11-BS70 [21] and MLV-016 [GenBank: NZ_ABGH00000000], are currently available in public databases.

AP200 has been previously reported to harbour the transposon Tn*1806*, carrying the erythromycin resistance determinant *erm*(TR), which is uncommon in *S. pneumoniae *[22]. The genome sequence yielded the whole sequence of Tn*1806 *and evidence for the presence of another exogenous element, a functional bacteriophage, designated ϕSpn_200.

## Results and Discussion

### General genome features

**Figure 1:**
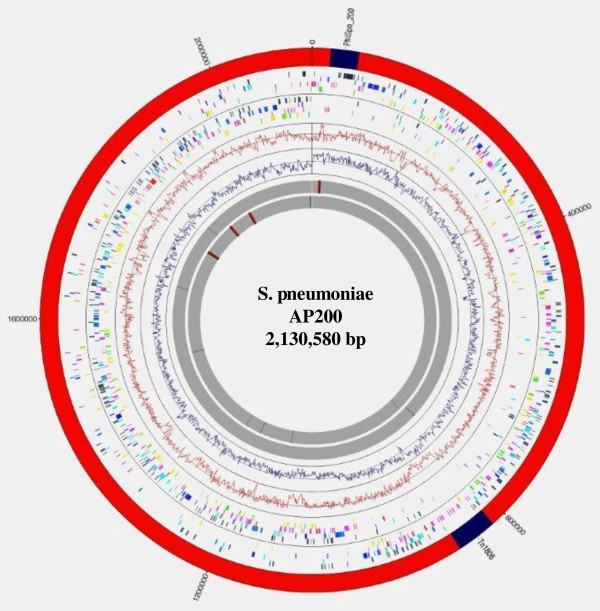
**Circular representation of *S. pneumoniae *AP200 chromosome**. Outer circle: distribution of the exogenous elements ϕSpn_200 and Tn*1806 *(dark blue). Second and third circles: predicted coding sequences on the plus and minus strand, respectively. Each circle has been divided in 4 rings according to the predicted functions:(from outer to inner ring) proteins poorly characterized, proteins involved in metabolism, proteins involved in information, storage and processing, proteins involved in cellular processes. Fourth circle: GC content. Fifth circle: GC deviation. Sixth and seventh circles: tRNA (dark green) and rRNA (red) on the plus and minus strand, respectively.

The AP200 chromosome is circular and is 2,130,580 base pair in length. The main features of the sequence are shown in Figure 1 and Table 1.The initiation codon of the *dnaA *gene, adjacent to the origin of replication *oriC*, was chosen as the base pair 1 for numbering the coding sequences. The overall GC% content is 39.5% but an unusual asymmetry in the GC skew is evident near positions 820,000-870,000, likely resulting from recent acquisitions through horizontal gene transfer. The genome carries 2216 coding sequences (CDS), 56 tRNA, and 12 rRNA genes grouped in four operons. Of the predicted CDSs, 1616 (72.9%) have a predicted biological known function; 145 (6.5%) are similar to hypothetical proteins in other genomes, and 455 (20.5%) have no substantial similarity to other predicted proteins.

**Table 1:** General characteristics of the *S. pneumoniae *AP200 genome.

Component of the genome	Property
Topology	Circular
Length	2,130,580 bp
G+C content	39.5%
Coding density	86.1%
Coding sequences	2,283
rRNA	12 genes in four sets
tRNA	56
CDS	2,216
conserved with assigned function	1,616 (72.9%)
conserved with unknown function	145 (6.5%)
nonconserved	455 (20.5%)
Average CDS length	828 bp
Exogenous elements	
ΦSpn_200	35,989 bp
Tn*1806*	52,457 bp
IS*1239*	10 copies
IS*1381*-ISSpn7	9 copies
IS*1515*	8 copies
ISSpn2 and IS*1167*	6 copies each
IS*630*, ISSpn1-3 and IS*1380*- ISSpn5	4 copies each
IS*1202*	1 copy
ISSpn_AP200_1 to ISSpn_AP200_7	1 to 3 copies

The AP200 genome contains approximately 170 kb that are not present in TIGR4 [GenBank: NC_010380], the first sequenced pneumococcal strain [23]. Besides two exogenous elements, such as the large Tn*1806 *transposon and a temperate bacteriophage designated ϕSpn_200, the extra regions include the type 11A capsular locus, the pilus islet 2 [24], and two metabolic operons (Additional file 1). Of the latter, one contains the genes of the arginine succinate pathway which is present in most pneumococci as a second alternative to the arginine deaminase pathway and the second likely contains genes for uptake, metabolism and excretion of sulphur containing amino-sugars. Three other operons containing uptake systems of unknown substrates are also present. Other regions of difference between TIGR4 and AP200 include the presence in the latter of a *Dpn*II restriction system and a double glycin-type bacteriocin gene (Additional file 1). The extent and type of genomic variation between AP200 and TIGR4 is in line with the genetic diversity found within this species by other studies comparing a series of pneumococcal genomes [21,25,26].

**Figure 2:**
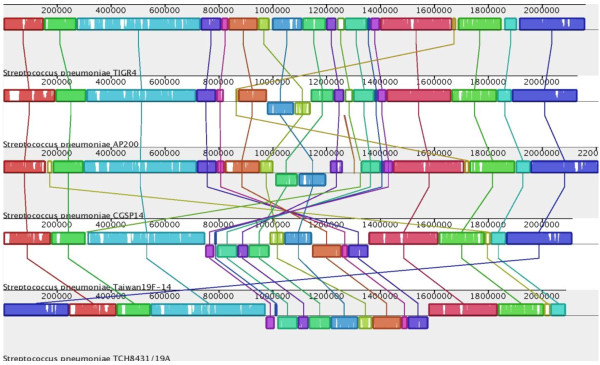
**Genome alignment of *S. pneumoniae *strains TIGR4, AP200, CGSP14, Taiwan 19F-14 and TCH8431/19A**. Each sequence of identically colored blocks represents a collinear set of matching regions linked across genomes. Regions that are inverted are shifted below a genome's center axis. Figure generated by Mauve, free/open-source software available from http://gel.ahabs.wisc.edu/mauve.

Comparison of the AP200 genome with TIGR4 revealed also a large chromosomal inversion of approximately 163 kb across the replication axis and involving the termination site (Figure 2). Large-scale inversions are typically driven by homologous recombination among repeated regions. The AP200 inversion borders fall within the coding sequences of PhtB and PhtD, two proteins which are part of the histidine-triad proteins family, characterized by the repeated histidine HxxHxH triad motif [27]. This family is composed of 4 proteins (PhtA, PhtB, PhtD, and PhtE) showing high sequence similarity. PhtB and PhtD, which are involved in AP200 chromosomal inversion, reach approximately 87% amino acids identity.

Chromosomal inversions are thought to be implicated in the rebalance of the chromosomal architecture when it is affected by insertions of large DNA regions, such as transposons, IS elements or prophages. In particular, it has been speculated that the chromosomal imbalance could be caused when large DNA fragments are inserted in one side of the replication axis [28], as in the case of AP200 genome, where the large exogenous elements resided in right of the replication axis. To date, the only pneumococcal genome described to carry a large chromosomal inversion is CGSP14 [28]. Also in CGSP14 the inversion occurs across the termination site but involves a different region (Figure 2). Inversions are present also in 2 recently sequenced pneumococcal genomes, Taiwan 19F-14 [GenBank: NC_012469] and TCH8431/19A [GenBank: NC_014251], although they have not been described (Figure 2). In these strains, the chromosomal inversions involve much larger regions. These observations suggest that the synteny of pneumococcal genome is not always conserved.

A striking feature of pneumococcal genomes is the over-distribution of IS elements [23,29]. AP200 contains 63 transposases and inactivated derivatives thereof http://www-is.biotoul.fr/is.html. In order of frequency, the insertion sequences present in the genome are IS*1239 *(10 copies), IS*1381*-ISSpn7 (9 copies), IS*1515 *(8 copies), ISSpn2 and IS*1167 *(6 copies each), IS*630*, ISSpn1-3 and IS*1380*-ISSpn5 (4 copies each), and IS*1202 *(one copy). Interestingly, for 3 of these families, the number and insertion site of the IS elements present in AP200 differ from those present in the other two serotype 11A, ST62 strains, SP11-BS70 [GenBank: NZ_ABAC00000000] and MLV-016 [GenBank: NZ_ABGH00000000], although the draft genome status of these two strains makes it impossible to carry out a complete comparison. Only 3 out of 8 IS*1515 *insertions, and only 2 out of 4 of the IS*1380*-ISSpn5 insertions are shared between AP200 and the other serotype 11A strains, while one of the IS*1239 *copies is present in AP200 only and is integrated in the *comC *gene, making AP200 unable to develop natural competence. The fact that the insertion sites for IS*1239*, IS*1380*, and IS*1515 *copies vary between ST62 strains suggests that these IS elements maintained their ability to transpose within the strains. In AP200, one copy of IS*1515 *is inserted within the *nanB *gene, producing a truncated Neuraminidase B. In addition to these known IS elements, other 7 non characterized elements are present in AP200 in a number of copy ranging from 1 to 3. These ISs have been named from ISSpn_AP200_1 to ISSpn_AP200_7.

Notably, AP200 shares with the other serotype 11A ST62 strains, an unique mutation in the 23S rRNA (T552C) that is not present in the other sequenced pneumococci. This mutation has also been confirmed by Sanger sequencing.

### Virulence factors

A plethora of virulence factors have been described in *S. pneumoniae *[30]. Among them, the most important is the polysaccharide capsule, shielding pneumococci from the host natural immune defense. The capsular serotype of AP200 was identified as 11A according to the Quellung reaction [31], but sequence analysis revealed that the capsular locus matched closely that of serotype 11D. In particular, AP200 showed only 3 nucleotide changes when compared to the 11D capsular locus of the reference strain 70/86 [GenBank: CR931656] [7]: two silent transitions in *wze *and *wchA*, respectively, and a G/A transition (G10118A) determining a change of a serine into an asparagine in the glycosyl transferase gene *wcrL*. Also the capsular locus of the two other ST62 serotype 11A strains, SP11-BS70 [21] and MLV-016 [GenBank: NZ_ABGH00000000], match with the 11D capsular locus. SP11-BS70, like AP200, has been repeatedly tested using the Quellung reaction by us and by the pneumococcal reference laboratory at the Statens Serum Institute, yielding consistently serotype 11A. From these results it appears that these ST62 isolates have a serotype 11A phenotype, but possess an 11D capsular locus. The same conclusion has been reached by Moon Nahm's laboratory examining the serotype 11A isolates obtained at the Centers for Disease Control and Prevention in Atlanta, GA (M. Nahm, personal communication).

Sequence differences between capsular locus 11A and 11D cluster mainly in the insertion sequence (IS*1202*) flanking the 5' end of the locus and in the *wcjE *gene, encoding a putative O-acetyl transferase. While the biochemical structure of the type 11A capsule is known [32], that of type 11D capsule has not been elucidated, therefore it is unclear which structural difference underlies the immunological difference. In addition, serotype 11D is quite rare, since no isolates of this serotype appear in the MLST database or in recent large datasets. On the other hand, recent findings indicate that serotype 11A has a high degree of genetic heterogeneity. A new pneumococcal serotype, designated 11E, has been recently discovered among isolates previously identified as serotype 11A, and has been found to be associated with a mutated or disrupted *wcjE *gene [10]. On the basis of these data and our results it appears that serotype 11 is genotypically variable and it is likely that its typing scheme will be reconsidered in the near future.

Most of the other pneumococcal virulence factors are surface-exposed proteins such as the choline-binding proteins (CBPs) and the LPXTG proteins. Ten different CBPs genes have been recognized in the genome of AP200, including *pspA *and *pspC*, which play an important role in pneumococcal pathogenicity [33,34]. Both these proteins are characterized by an extensive polymorphism, likely reflecting the immunological selective pressure to which they are exposed. According to the classification of Hollingshead *et al*. [35], that defines 6 immunologically-relevant monophyletic groups (clades) on the basis of the divergence of the PspA central region, AP200 PspA belongs to clade 3. Similarly, the PspC protein has been divided into 11 major groups due to unique sequence blocks [36]. According to this classification, AP200 PspC corresponds to PspC3.

The LPXTG family includes proteins anchored to the peptidoglycan cell wall by the action of a sortase transpeptidase that recognises the motif LPXTG. Pili, recently discovered in pneumococci, are composed of LPXTG-type protein subunits, and can be of 2 types, encoded by 2 different islets, PI-1 and PI-2 [24,37]. AP200 carries PI-2, that is found in 20% of pneumococci only and has been demonstrated to mediate adherence to the epithelial cells of the respiratory tract [24]. The PI-2 region in AP200 is identical to that of serotype 1 PN110 strain [24], being flanked by the *hemH *and *pepT *genes, but is contained in the 163 kb inversion. Of the two other sequenced serotype 11A ST62 strains, only SP11-BS70 carries PI-2. A recent investigation on the prevalence of PI-2-carrying pneumococcal isolates in Atlanta, USA, highlighted the increase of serotypes carrying PI-2 among emerging non-PCV7 serotypes, including serotype 11A [38].

Four large surface zinc metalloproteinases have been described in *S. pneumoniae*, including the IgA protease, which cleaves human IgA1 [39], the ZmpC proteinase, which cleaves human matrix metalloproteinase 9 [40] and ZmpB and ZmpD, whose substrates have not yet been identified [41]. The zinc metalloproteinases are involved in virulence and possess antigenic properties [42]. AP200 carries three of them, *iga*, *zmpB *and *zmpC*, lacking *zmpD*.

### Mobile genetic elements of AP200

#### Tn*1806*

**Figure 3:**
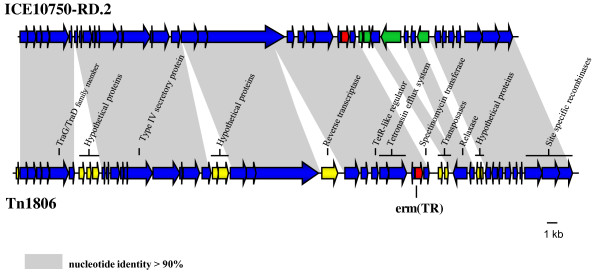
**Schematic representation of Tn*1806 *of *S. pneumoniae *AP200, in comparison with ICE10750 RD-2 of *S. pyogenes***. The *erm*(TR) gene is indicated by a red arrow. Blue arrows indicate shared ORFs between the 2 elements. Yellow arrows indicate the ORFs uniquely present in Tn*1806 *while green arrows indicate those uniquely present in ICE10750 RD-2. Shaded areas between the elements indicate a nucleotide identity greater than 90%. The proteins of Tn*1806 *indicated in the figure are described in the text.

The Tn*1806 *transposon represents the sole *erm*(TR)-carrying genetic element reported in *S. pneumoniae *to date, and only a partial sequence was published by our group in 2008 [22]. Tn*1806 *is 52,457 bp in size, smaller than the size previously estimated by PCR mapping [22], has a GC content of 31.1%, and comprises 49 ORFs. The chromosomal insertion site (*hsdM *gene) of Tn*1806 *is characterized by the duplication of 3 nucleotides (GGG) representing the target sequence for the integration [22]. Although various proteins related to mobilization are present, such as a TraG/TraD protein, a Type IV secretory protein, a relaxase and 3 recombinases at the right end (Figure 3 and Additional file 2), conjugation experiments have failed to show transferability of Tn*1806 *to other strains [22]. Other putative antibiotic resistance genes are present in Tn*1806 *in the region flanking *erm*(TR), such as the two components of a tetronasin ABC-type efflux system and a spectinomycin phosphotransferase. A TetR family transcriptional regulator is located upstream of the tetronasin efflux system, likely being involved in its regulation [43,44].

Tn*1806 *shows an overall similarity with the *erm*(TR)-carrying genetic element described in *Streptococcus pyogenes *MGAS10750, named ICE10750 RD-2 [45]. ICE, Integrative and Conjugative Element, identifies a new classification nomenclature, grouping self-transmissible genetic elements previously designated as transposons, conjugative transposons, genomic islands and plasmids, sharing a common mechanism of horizontal transfer via site-specific recombination [46]. In this broad definition, also Tn*1806 *can be considered an ICE. Tn*1806 *is approximately 4 kb larger than ICE 10750-RD.2 due to the presence of additional regions (Figure 3). Starting from the 5' end of the element, Tn*1806 *contains 3 additional ORFs homologous to hypothetical proteins of the chimeric element RD1 of *S. pyogenes *MGAS6180 [47], 2 ORFs homologous to hypothetical proteins contained in the plasmid pAPRE01 of *Anaerococcus prevotii *DSM20548, and a retron-type reverse transcriptase inserted inside the adenine-specific DNA methylase gene. In addition, in Tn*1806 *downstream *erm*(TR), 2 transposases replace a cytidine deaminase and a Zn-dependent hydrolase present in ICE 10750-RD.2, while 2 hypothetical proteins replace an ORF, which is predicted to encode a death on curing protein, part of a toxin-antitoxin system (Figure 3). The antibiotic-resistance region, including the *erm*(TR) flanking genes, is present in ICE10750 RD-2 [45] as well as in other *S. pyogenes erm*(TR)-carrying elements recently described [48].

**Figure 4:**
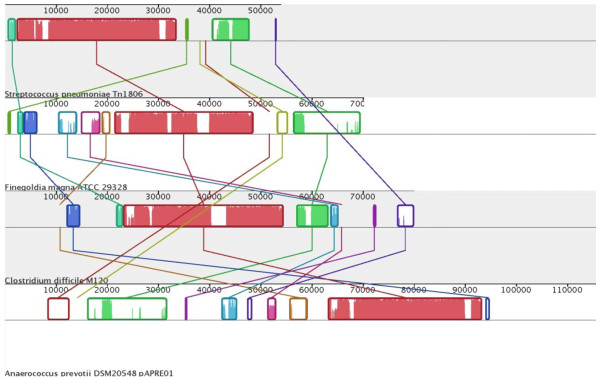
**Nucleotide alignment of Tn*1806 *with the predicted genetic elements of *F. magna *ATCC29328 and *C. difficile *M120, and with the plasmid pAPRE01 of *A. prevotii *DSM20548**. Each sequence of identically colored blocks represents a collinear set of matching regions. Figure generated by Mauve, free/open-source software available from http://gel.ahabs.wisc.edu/mauve.

Comparative nucleotide analysis with current databases revealed that Tn*1806 *shows large regions of homology with other putative genetic elements present in the sequenced genomes of different bacterial species, including *Finegoldia magna *ATCC 29328 [GenBank: AP008971] [49] and *Clostridium difficile *M120 [GenBank: FN665653], and with pAPRE01, a plasmid of *A. prevotii *DSM20548 [GenBank: CP001709]. All these species are anaerobic opportunistic pathogens; *F. magna *and *A. prevotii *share the same ecological niche, i.e. the oral cavity, with *S. pneumoniae *and *S. pyogenes*, while *C. difficile *is part of the intestinal microflora. The genetic elements of these three anaerobic species share a high nucleotide identity (88-95%) especially with the leftmost part of Tn*1806 *(Figure 4). Sequences with similarity to Tn*1806 *have been found also in the incomplete genome of *Ureaplasma urealyticum *serovar 9 ATCC 33175 [GenBank: NZ_AAYQ02000002] and in other incomplete genomes belonging to *Anaerococcus *spp. and *Peptoniphilus *spp. All these genetic elements share large fragments, with insertions/deletions or replacement of different modules that probably confer element-specific features. Modules can contain different accessory genes: one example is represented by the antibiotic-resistance region that is present in Tn*1806 *and ICE10750 RD-2, but is missing in the other genetic elements. In *F. magna*, this region is replaced by a module of similar size including multidrug ABC transporter proteins (Additional file 3). These elements, carried by different bacterial species, likely diversify and evolve through the reciprocal shuffling of regions in putative hot spots; the diversity likely reflects the adaptation to different niches and/or to the antibiotic selective pressure.

#### ϕSpn_200 prophage genome

The second exogenous region identified in AP200 corresponds to a prophage designated ϕSpn_200. The ϕSpn_200 genome is 35,989 kb in size with a GC content of 39.3%, which is consistent with that of *S. pneumoniae*. ϕSpn_200 is inserted between the adenylosuccinate synthetase and the tRNA-specific adenosine deaminase genes. Sequence analysis of the junctions between the ϕSpn_200 genome and the host chromosome revealed the presence of a 21-bp duplication (5'- CTTTTTCATAATAATCTCCCT -3'), likely derived from the recombination between the bacterial (*attB*) and the phage (*attP*) attachment sites. The confirmation that the 21-bp region corresponds to the *attP *site was obtained by sequencing the DNA of the phage circular forms.

**Figure 5:**
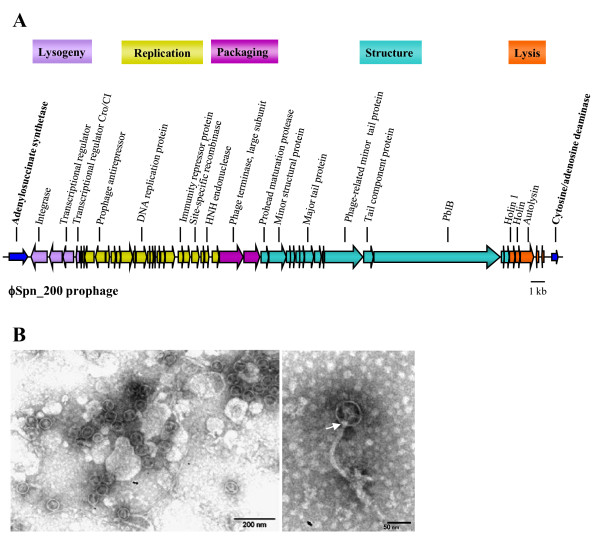
**Characterization of ϕSpn_200**. A) Genomic organization of ϕSpn_200 prophage. The colors of the ORFs (arrows) of ϕSpn_200 are in accordance with their predicted function: violet refers to genes involved in lysogeny, yellow to genes involved in replication/immunity, fuchsia to genes involved in packaging, turquoise to genes involved in the structure and orange to genes involved in lysis. Some of the proteins indicated are described in the text. Blue arrows at both ends of the prophage indicate the ORFs of the host chromosome. B) Detection of phage particles in the supernatant of strain AP200 induced to lysis by mitomycin C. Electron micrographs show: several viral particles (left) and a single phage particle with a collar structure (arrow) and a slightly bent tail (right).

The genome of ϕSpn_200 includes a total of 47 ORFs organized into five modules: the lysogeny, the replication, the packaging, the structural, and the lytic modules (Figure 5A). Such modular organization, especially the presence of closely arranged lysogeny-related genes, resembled that of the *Siphoviridae *family infecting low-GC content Gram-positive bacteria [50]. The predicted ORFs were compared with sequences from protein databases and the regions of homology of the ϕSpn_200 genome are described in detail in the Additional file 4.

The lysogeny module is located immediately downstream of the left-end *att *site; it is composed of the integrase, belonging to the family of tyrosine recombinases, the Cro/CI-like transcriptional regulator and the repressor involved in suppression of the phage lytic cycle (Figure 5A). The second module carries genes with regulatory functions implicated in the replicative processes. The third module includes genes implicated in the packaging of the phage genome concatemers into the empty capsid shell, such as the large terminase gene. The structural region encodes the morphogenetic proteins involved in the head and tail assembly. Among these proteins, it is noteworthy the presence of PblB that corresponds to the phage tail fiber, involved in tail/host recognition. This protein is also considered a phage-encoded virulence factor [51]. In *Streptococcus mitis*, PblB is carried by the bacteriophage SM1 and together with PblA, a protein that is missing in ϕSpn_200, it can enhance binding of the microorganism to platelets [51,52]. No other potential virulence factor was identified in ϕSpn_200, but it must be considered that no function was assigned to 28 out of 47 phage ORFs. The last phage module includes genes implicated in cell lysis and phage progeny release, such as those encoding lysin and holin proteins. Holin acts creating holes in the cell wall, thereby allowing lysin to enter the periplasm and begin cell lysis.

**Figure 6:**
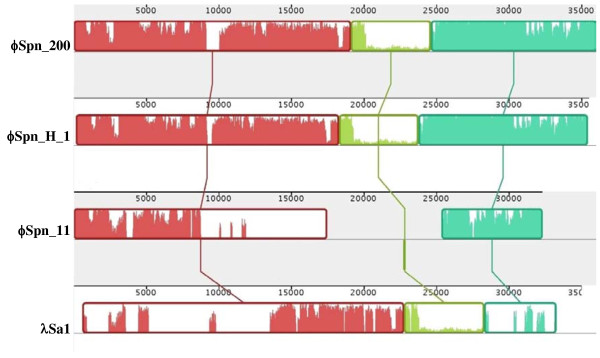
**Nucleotide alignment of ϕSpn_200 with ϕSpn_H_1 (prophage present in *S. pneumoniae *Hungary 19A-6, GenBank: **CP000936), **ϕSpn_11 (prophage present in *S. pneumoniae *SP11-BS70, GenBank: **NZ_ABAC00000000) **and with λSa1 (prophage present in *S. agalactiae *2603 V-R, GenBank: **NC_004116). Each sequence of identically colored blocks represents a collinear set of matching regions. Figure generated by Mauve, free/open-source software available from http://gel.ahabs.wisc.edu/mauve.

An almost identical prophage, inserted in the same chromosomal region at the identical *attB *attachment site, is present in the newly sequenced *S. pneumoniae *strain Hungary19A-6 [GenBank: CP000936], and in the draft genomes of CDC1873-00 [GenBank: NZ_ABFS01000005] and SP14-BS69 [GenBank: NZ_ABAD01000021] (Figure 6). Interestingly, a prophage inserted in the same site of ϕSpn_200, is present also in the SP11-BS70 genome, named ϕSpn_11 [53]. ϕSpn_11 and ϕSpn_200 represent different phages although they share the integrase and the following ORF of the lysogeny module, 12 out of 21 genes of the replication module and all the lytic genes (Figure 6). Comparative analysis revealed that ϕSpn_200 showed various degree of similarity with other streptococcal prophages. The ϕSpn_200 packaging and structural modules are highly similar to the corresponding regions of phage LambdaSa2 of *Streptococcus agalactiae *2603 V/R [54], with an amino acid identity ranging from 53 to 92% (Figure 6). The presence in ϕSpn_200 of functional modules, carried also by a different phage, supports the modular theory of phage evolution [50] according to which the diversification of phages genomes resides mainly on the exchange of entire modules between different phage groups. Indeed, in pneumococcal phages the exchanging unit could consist also in a single gene [53], as it was the case suggested by the homology of single genes of the replication module of ϕSpn_200 with the corresponding genes of phage MM1 of *S. pneumoniae *[55], of phage SM1 of *S. mitis *[56] and LambdaSa2 of *S. agalactiae *2603 V/R [54].

According to a recently published prophage typing system [57], the pneumococcal phages can be classified into three main groups, of which group 1 is the most abundant. On the basis of nucleotide homologies, ϕSpn_200 can be assigned to group 1.

#### Electron microscopic characterization and infection activity of ϕSpn_200

Concentrated supernatants of mitomycin-induced *S. pneumoniae *AP200 cultures were examined by transmission electron microscopy. Ultrastructural analysis revealed the presence of phage particles consisting of a small isometric head with a diameter of 56 ± 2 nm and a long flexible tail of 156.8 ± 2 nm, characteristics belonging to the *Siphoviridae *family [58] (Figure 5B). A collar structure was observed at the position where head and tail meet (Figure 5B). Since only one prophage was detected in the genome of AP200, we concluded that the phage observed by electron microscopy was ϕSpn_200.

The infection activity of ϕSpn_200 was tested on the pneumococcal strain Rx1 [59]. Results obtained demonstrated that ϕSpn_200 induced the formation of lysis plaques on the Rx1 culture plates (Additional file 5).

## Conclusions

The number of sequences of bacterial genomes has been rapidly increasing in the last years thanks to the use of new technologies, such as the high-throughput Roche 454 pyrosequencing [60,61]. *S. pneumoniae *serotype 11A is becoming an emergent serotype in the post-PCV7 era and data concerning its genetic characteristics can be of importance for future vaccines. The reasons determining the increase in the incidence of pneumococcal infections due to non vaccine-serotypes, including serotype 11A, are complex and not yet fully understood. Multiple factors could take part in this phenomenon, such as geographical and temporal trends, the prevalence of these serotypes in the community, the ability to evade host defenses, the acquisition of new genetic material that could potentially increase their invasive capacity or their resistance to antibiotics [62].

In this study, the entire genomic sequence of *S. pneumoniae *AP200, belonging to serotype 11A and ST62, has been obtained. Sequence analysis revealed chromosomal rearrangements and horizontal gene transfers. A large chromosomal inversion across the replication axis was found: it is likely that this inversion originated to maintain the genome stability affected by horizontal gene transfer events, as suggested by Ding *et al*. [28]. The presence of large genomic inversions is a phenomenon observed in other streptococcal species, where it could contribute to generate chromosomal shuffling and create novel genetic pools [63-65].

Horizontal gene transfer events involved mainly two mobile elements, the *erm*(TR)-carrying genetic element Tn*1806 *and the functional prophage ϕSpn_200. The modular organization recognized inside the two exogenous elements, and their similarity to other elements of different bacterial species, confirm that they have undergone frequent DNA exchanging events, that appear to be the major contributors to the overall diversity of the genome of *S. pneumoniae *AP200.

Although the availability of complete pneumococcal genomes cannot provide a full explanation for the evolution and spread of a particular serotype or clone, it can contribute information on the pathogenic potential of this important microorganism. Regarding AP200, the presence of pilus islet 2 could confer a selective fitness advantage, mediating adherence to the nasopharingeal epithelium and could represent a target for future vaccines [24,38]. In addition, the presence of the transposon Tn*1806*, conferring erythromycin-resistance, is an advantage to the microorganism in view of the large use of macrolides in the community. Finally, in AP200 the discrepancy between the serotyping result and the sequence of the capsular locus deserves further investigations, also in view of the increasing use of PCR-based methods for serotype determination.

## Methods

### Bacterial strain

*S. pneumoniae *AP200 was isolated from the cerebrospinal fluid of an adult patient with meningitis in 2003 [22]. AP200 was found to belong to serotype 11A and to ST62, although previously it had been erroneously attributed to a different ST. ST62 is the predicted founder of CC62, to which most serotype 11A isolates belong http://spneumoniae.mlst.net/. AP200 is resistant to erythromycin, with a MIC of 1 μg/ml, and shows inducible resistance to clindamycin due to the presence of the *erm*(TR) resistance gene [22].

### Sample Preparation and High-density Pyrosequencing

Genomic DNA of AP200 (4 ug), prepared using the Cell and Blood Culture DNA Midi kit (Qiagen, Valencia, CA), was fragmented by nitrogen nebulization for 1 minute at the pressure of 45 psi. Fragmented DNA was purified using silica spin-columns (MinElute PCR purification kit, Qiagen, Valencia, CA) and subsequently analyzed by Agilent Bioanalyzer 2100 with the DNA 1000 Kit (Agilent Technologies, Palo Alto, CA, USA) to check the average fragment size. The double- stranded fragmented DNA was prepared as reported in Roche-454 Library Preparation Manual to obtain the ssDNA library. The sample was analyzed with Agilent Bioanalyzer 2100 and the mRNA Pico Kit (Agilent Technologies), and was fluorometrically quantitated by RiboGreen RNA Quantitation Kit (Invitrogen Inc., Carlsbad, California). A second DNA library (insert size 2000-2500 bp) was prepared starting from 3 ug of total genomic DNA to perform Paired-Ends sequencing, following the Roche-454 Paired End Library Preparation Manual. The samples prepared for the standard shotgun and for the Paired-Ends sequencing were sequenced by means of Genome Sequencer 454 FLX [66].

### Sequencing Data analysis

A total of 263,671 high-quality sequences and 37,704,248 bp were obtained with a 17-fold coverage of the genome. The 454 de Novo Assembler software was used to assemble the sequences that were read. This first automatic step produced 130 contigs, where 91 were large contigs with a maximum size of 149,967 bp. The de novo assembly created 8 scaffolds for a total of 2,107,179 bp, the largest scaffold's size being 1,176,929 bp. A manual check of every added sequence read to confirm the correct assembly was performed. Gaps between and inside the 8 scaffolds, due to difficult assembly of repetitive DNA and complex regions, have been solved using long PCR strategy and Sanger sequencing. A manual inspection of the final assembly was required. Since homopolymeric stretches into the genome can determine a high probability of frameshift error during the assembly of the sequence, potential errors were checked by visual inspection of the sequences read.

### Genome annotation and comparison

The generated sequences were annotated identifying coding genes by cross prediction from the FGENESB package http://www.softberry.com/, the GeneMark program [67] and the GLIMMER program [68]. We considered an open reading frame (ORF) prediction to be good when it was identified by each of the three prediction tools. Discrepant ORFs were manually verified by the Artemis viewer [69] and by identification of putative ribosomal binding sites. Each gene was functionally classified by assigning a cluster of orthologous group (COG) number or a Kyoto encyclopedia of genes and genomes (KEGG) number, and each predicted protein was compared against every protein in the non- redundant (nr) protein databases http://ncbi.nlm.nih.gov. In order to associate a function with a predicted gene, we used a minimum cut-off of 30% identity and 80% coverage of the gene length, checking at least two best hits among the COG, KEGG, and non- redundant protein databases. The rRNA genes were identified by the FGENESB tool on the basis of sequence conservation, while tRNA genes were detected with the tRNAscan-SE program. The BLASTp algorithm was used to search for protein similarities with other pneumococcal genomes or deposited sequences referred in the present study, following these criteria: >50% similarity at the amino acid level and >50% coverage of protein length.

### Phage characterization

AP200 was grown in BHI broth at 37°C to achieve a turbidity corresponding to OD_620 _0.2-0.3. Mytomycin C (Sigma-Aldrich, St. Louis, MO) was added to a final concentration of 0.1 μg/ml and the culture was incubated until lysis occurred, as shown by a decrease in turbidity. Cellular debris was pelleted at 16000 *g *for 15 min. The induced supernatant was filtered through a 0.44-μm pore size filter (Millipore, Billerica, MA). For negative staining, the filtered supernatant was ultracentrifuged at 100,000 *g *for 2 h at 4°C. Suspensions of the pellet were placed on Formvar-carbon coated 400 mesh copper grids for 10 s, wicked with filter paper and placed on a drop of 2% sodium phosphotungstate, pH 7.00, for 10 s, wicked again and air-dried. Negatively stained preparations were observed with a Philips 208 electron microscope at 80 kV.

To obtain phage DNA, the phage pellet was lysed with sodium dodecyl sulfate (0.5%), EDTA (10 mM) and proteinase K (500 μg/ml) for 2 h at 37°C. Phage DNA was precipitated with a 10% volume of 3 M NaOAc (pH 5.2) and 2 volumes of ethanol at -70°C for 2 h, washed with 70% ethanol and resuspended in deionized H_2_O. In order to demonstrate the circularization of the excised prophage, a PCR assay using the phage DNA as template and divergent primers pair (FR9 5'- CTAGACTTGCGATAGCAGTTACC- 3' and FR10 5'- GCTTGAACAATTAAGCCAAGCG-3') designed on the opposite ends of the prophage sequence, was carried out. The PCR product was purified and submitted to sequencing analysis using a Perkin-Elmer ABI 377 DNA sequencer (PE Applied Byosystem).

To demonstrate phage activity, a plaque assay was performed. Briefly, 0.1 ml of filtered induced supernatant was pre-incubated with 0.9 ml of the pneumococcal indicator strain Rx1 [60] at about 10^8 ^cells/ml for 30 min at 37°C [70]. 0.1 ml of this adsorption mix was added to 3 ml of 2% blood soft agar, poured on a plate containing a layer of bottom agar and incubated overnight at 37°C.

### Nucleotide sequence accession numbers

The AP200 genome sequence was submitted to the GenBank database [GenBank: CP002121]. The nucleotide sequence of Tn*1806 *was deposited as an update of GenBank accession number [GenBank: EF469826].

## Abbreviations

ORF: Open Reading Frame; CDS: protein coding sequence.

## Authors' contributions

RC performed the analysis of genetic elements, the phage induction experiments and drafted the manuscript. RJPB, MI and GC performed the bioinformatic analysis and participated in genome comparison. MDG and FI participated in the analysis and comparison of the exogenous genetic elements. ER performed DNA preparation and generated the 454 sequencing data. FS and MM carried out the ultrastructural characterization of phage particles. LM participated in the genome comparison. GDB participated in the design of the study, its coordination and helped in revising the manuscript. MRO participated in the design of the study, carried out the genome comparison and helped in writing the manuscript. AP participated in the design of the study, its coordination and finalized the manuscript. All authors read and approved the final manuscript.

## Supplementary Material

Additional file 1 **Table S1**. AP200 chromosomal additional regions with respect to TIGR4 genome. This table summarizes the regions of diversity between AP200 and TIGR4 genomes. Click here for file

Additional file 2 **Table S2**. Comparative analysis of the genes from Tn*1806 *with proteins included in the databases. This table summarizes the homologies of the ORFs of Tn*1806 *with proteins included in current databases. Click here for file

Additional file 3 **Figure S3**. Schematic representation of Tn*1806 *of *S. pneumoniae *AP200, in comparison with the predicted genetic element of *F. magna *ATCC29328. This figure describes in detail the regions of similarity between the two genetic elements. Click here for file

Additional file 4 **Table S4**. Comparative analysis of the genes from ϕSpn_200 with proteins included in the databases. This table summarizes the homologies of the ORFs of ϕSpn_200 with proteins included in current databases. Click here for file

Additional file 5 **Figure S5**. Phage plaque assay using the *S. pneumoniae *indicator strain Rx1. This figure shows the Rx1 lawn lysis due to ϕSpn_200 activity. Click here for file
