# Supplementary material for: Complete genome sequence of a serotype 11A, ST62 Streptococcus pneumoniae invasive isolate
Source: BMC Microbiol. 2011 Feb 1;11:25. doi: 10.1186/1471-2180-11-25 (PMC3055811; doi:10.1186/1471-2180-11-25)
Supplement: Additional file 1 — Table S1. AP200 chromosomal additional regions with respect to TIGR4 genome. This table summarizes the regions of diversity between AP200 and TIGR4 genomes. [file 1471-2180-11-25-S1.DOC]

# Supplementary Table S1. AP200 chromosomal additional regions with respect to TIGR4 genome

| **ORF** | **Size (bp)** | **Content** | **Other pneumococcal genomes containing the regions** |
| --- | --- | --- | --- |
| SPAP_0025 - SPAP_0071 | 35,989 | φSpn_200 prophage | Hungary 19A-6, CDC1073 and SP14-BS69 |
| SPAP_0156 - SPAP_0157 | 2,616 | Argininosuccinate synthase and arginine succinate lyase | SP11-BS70, MLV-016, D39, R6, CDC1087-00, CCRI 1974M2, CCRI 1974, JJA, Hungary19A-6 and 70585 |
| SPAP_0158 - SPAP_0170 | 9,294 | Transcriptional regulator and 12 hypothetical proteins | SP11-BS70, MLV-016 and most sequenced pneumococci |
| SPAP_0373 - SPAP_0390 | 18,542 | Type 11A capsule operon | SP11-BS70, MLV-016 |
| SPAP_0853 - SPAP_0855 | 2,957 | 2 transposases and ABC transporter | SP11-BS70, MLV-016, SP6-BS73 and CDC1087-00 |
| SPAP_0864 - SPAP_0912 | 52,457 | Tn*1806* | None |
| SPAP_1132 - SPAP_1147 | 10,566 | 16 genes in multiple transcriptional units | SP11-BS70, MLV-016, G54 and P1031 |
| SPAP_1157 - SPAP_1161 | 4,079 | 4 hypothetical proteins and a exonuclease V | SP11-BS70, MLV-016, and most sequenced pneumococci |
| SPAP_1189 - SPAP_1196 | 7,475 | Type II pilus islet | SP11-BS70, Taiwan19F-14, CDC1087-00, Canada MDR_19F Ctg345, TCH8431/19A, Canada MDR_19A Ctg690 |
| SPAP_1238 | 2,930 | Hypothetical protein | SP11-BS70, MLV-016, D39, R6, G54 and CGSP14 |
| SPAP_1767 - SPAP_1770 | 3,669 | Hypothetical protein, permease, ATP binding protein, and a transcriptional regulator | SP11-BS70, MLV-016, SP14-BS69, SP18-BS74, SP195, SP9-BS68 and P1031 |
| SPAP_1789 - SPAP_1806 | 12,765 | 18 genes including integral membrane proteins, 3 genes involved in glycosaminoglycan degradation and a phosphotransferase system (PTS) | SP11-BS70, MLV-016, SP23-BS72, 70585, CGSP14, CCRI 1974M2, CCRI 1974, JJA, SP3-BS71, Hungary19A-6, CDC1087-00, SP195 and ATCC 700669 |
| SPAP_1845 - SPAP_1846 | 1,650 | *Dpn*II restriction endonuclease and a DNA methylase | SP11-BS70, MLV-016, G54, CDC0288-04, Hungary19A-6, P1031, SP18-BS74 and 70585 |
| SPAP_1880 - SPAP_1884 | 2,514 | ABC transporter system | SP11-BS70, MLV-016, P1031, CCRI 1974M2, CCRI 1974, SP195, SP9-BS68, CDC0288-04, SP14-BS69, CDC1873-00, CDC3059-06, SP19-BS75, JJA and ATCC 700669 |
| SPAP_1923 - SPAP_1926 | 3,736 | Hypothetical proteins and a SAM-dependent methyltransferase | SP11-BS70, MLV-016, G54, TCH8431/19A, Taiwan19F-14, SP6-BS73, CDC0288-04, SP23-BS72, SP19-BS75, SP3-BS71, SP18-BS74 and CDC1087-00 |
| SPAP_1999 - SPAP_2001 | 2,128 | Double**-**glycine-type bacteriocin, transposase and a transcriptional regulator | SP11-BS70, MLV-016 |
