# Supplementary material for: Complete genome sequence of a serotype 11A, ST62 Streptococcus pneumoniae invasive isolate
Source: BMC Microbiol. 2011 Feb 1;11:25. doi: 10.1186/1471-2180-11-25 (PMC3055811; doi:10.1186/1471-2180-11-25)
Supplement: Additional file 2 — Table S2. Comparative analysis of the genes from Tn1806 with proteins included in the databases. This table summarizes the homologies of the ORFs of Tn1806 with proteins included in current databases. [file 1471-2180-11-25-S2.DOC]

**Supplementary Table S2.** Comparative analysis of the genes from Tn*1806* with proteins included in the databases. The Start and the Stop codons of the ORFs are referred to EF469826.

| **ORF** | **Start** | **Stop** | **Size**  **(amino acid)** | **Putative function** | **BLASTP best match** | **Accession no.** | **% Amino acid**  **identity**  **(% similarity)** |
| --- | --- | --- | --- | --- | --- | --- | --- |
| *orf1* | 792 | 1037 | 81 | - | Hypothetical cytosolic protein  (*Geobacillus sp*. G11MC16) | ZP_03148513 | 52 (71) |
| *orf2* | 1119 | 1892 | 257 | Replication initiator protein A (RepA) N-terminal domain protein | Hypothetical protein  (*Anaerococcus hydrogenalis* DSM 7454) | ZP_03303731 | 98 (99) |
| *orf3* | 1889 | 2716 | 275 | DNA replication protein | Chromosomal replication initiator protein DnaA  (*Finegoldia magna* ACS-171-v-Col3) | ZP_07269275 | 95 (98) |
| *orf4* | 2709 | 3194 | 161 | - | Hypothetical protein  (*Finegoldia magna* ATCC53516) | ZP_06947293 | 99 (99) |
| *orf5* | 3191 | 3925 | 244 | Phage antirepressor protein | BRO family, N-terminal domain protein (*Finegoldia magna* ACS-171-V-Col3) | ZP_07269286 | 99 (99) |
| *orf6* | 3903 | 5714 | 603 | Type IV secretory pathway protein VirD4 | TraG/TraD family (*Streptococcus pyogenes* MGAS10750) | YP_603177 | 96 (98) |
| *orf7* | 5797 | 6262 | 156 | Transcriptional regulator | Transcriptional regulator (*Eubacterium yurii* subsp. *margaretiae* ATCC 43715) | ZP_07454657 | 57 (72) |
| *orf8* | 6536 | 6730 | 64 | - | Putative plasmid transfer factor (*Finegoldia magna* ATCC 29328) | YP_001692276 | 100 (100) |
| *orf9* | 6794 | 7312 | 172 | - | Putative cytoplasmic protein (*Streptococcus pyogenes* MGAS6180) | YP_280557 | 92 (97) |
| *orf10* | 7350 | 7772 | 140 | - | Hypothetical protein (*Peptoniphilus* sp. oral taxon 836 str. F0141) | ZP_07095057 | 99 (100) |
| *orf11* | 7840 | 8457 | 205 | - | Putative cytoplasmic protein (*Streptococcus pyogenes* MGAS6180) | YP_280555 | 98 (99) |
| *orf12* | 8679 | 8990 | 103 | - | Hypothetical protein  (*Finegoldia magna* ATCC29328) | YP_001692273 | 95 (96) |
| *orf13* | 8992 | 9207 | 71 | - | Hypothetical protein ANHYDRO_00084 (*Anaerococcus hydrogenalis* DSM 7454) | ZP_03303695 | 100 (100) |
| *orf14* | 9218 | 10081 | 287 | - | Hypothetical protein (*Anaerococcus prevotii* DSM 20548) | YP_003142387 | 99 (100) |
| *orf15* | 10091 | 10381 | 96 | - | Hypothetical protein MGAS10750_Spy1688 (*Streptococcus pyogenes* MGAS10750] | YP_603182 | 98 (100) |
| *orf16* | 10383 | 10793 | 136 | - | Hypothetical protein MGAS10750_Spy1689 (*Streptococcus pyogenes* MGAS10750) | YP_603183 | 98 (100) |
| *orf17* | 10684 | 13107 | 807 | Type IV secretory pathway protein VirB4 | Hypothetical protein FMG_0960 (*Finegoldia magna* ATCC 29328) | YP_001692268 | 99 (100) |
| *orf18* | 13115 | 15694 | 859 | Cell wall-associated idrolases (invasion-associated proteins) | NlpC/P60 family protein (*Finegoldia magna* ACS-171-V-Col3) | ZP_07269265 | 95 (97) |
| *orf19* | 15709 | 15948 | 79 | - | Hypothetical protein (*Finegoldia magna* ACS-171-V-Col3) | ZP_07269262 | 98 (100) |
| *orf20* | 15935 | 17518 | 527 | - | Copper amine oxidase N-terminal domain protein (*Anaerococcus prevotii* DSM 20548) | YP_003142393 | 96 (97) |
| *orf21* | 17533 | 18399 | 288 | - | Putative bacteriocin (*Finegoldia magna* ATCC 29328) | YP_001692265 | 97 (98) |
| *orf22* | 18511 | 19086 | 191 | - | Hypothetical protein Apre_1820 (*Anaerococcus prevotii* DSM 20548) | YP_003142395 | 93 (97) |
| *orf23* | 19079 | 20062 | 327 | - | Hypothetical protein (*Finegoldia magna* BVS033A4) | ZP_07321273 | 91 (96) |
| *orf24* | 20155 | 21876 | 573 | Topoisomerase IA | DNA topoisomerase type IA central domain protein (*Anaerococcus prevotii* DSM 20548) | YP_003142397 | 96 (98) |
| *orf25* | 21869 | 22834 | 321 | Site-specific DNA methylase | DNA (cytosine-5-)-methyltransferase (*Peptoniphilus* sp. oral taxon (836 str. F0141) | ZP_07094115 | 96 (97) |
| *orf26* | 22821 | 29117 | 2098 | DNA and RNA methylase | Superfamily II DNA and RNA helicase (*Streptococcus pyogenes* MGAS10750) | YP_603192 | 96 (98) |
| *orf27* | 29673 | 31208 | 511 | Retron-type reverse transcriptase | Reverse transcriptase (RNA-dependent DNA polymerase) (*Finegoldia magna* BVS033A4) | ZP_07320521 | 98 (99) |
| *orf28* | 32849 | 33505 | 218 | - | Hypothetical protein (*Streptococcus pyogenes*) | CAQ56284 | 98 (99) |
| *orf29* | 34058 | 34690 | 210 | TetR family transcriptional regulator | Transcriptional regulator, TetR family (*Streptococcus pyogenes* MGAS10750) | YP_603194 | 100 (100) |
| *orf30* | 34775 | 35656 | 293 | Tetronasin resistance ATP-binding protein | Tetronasin resistance ATP-binding protein (*Streptococcus pyogenes* MGAS10750) | YP_603195 | 100 (100) |
| *orf31* | 35589 | 37295 | 568 | Tetronasin resistance transmembrane protein | Tetronasin resistance transmembrane protein  (*Streptococcus pyogenes* MGAS10750) | YP_603196 | 100 (100) |
| *orf32* | 37876 | 38082 | 68 | - | Hypothetical protein MGAS10750_Spy1704 (*Streptococcus pyogenes* MGAS10750) | YP_603197 | 100 (100) |
| *orf33* | 38029 | 38760 | 243 | *erm*(A) subclass *erm*(TR) | rRNA adenine N-6-methyltransferase  (*Streptococcus pyogenes* MGAS10750) | YP_603198 | 100 (100) |
| *orf34* | 38977 | 39501 | 174 | Spectinomycin phosphotransferase | Spectinomycin phosphotransferase (*Streptococcus pyogenes* MGAS10750) | YP_603199 | 100 (100) |
| *orf35* | 40522 | 40947 | 141/366 | Transposase | Transposase (*Finegoldia magna* ATCC 53516) | ZP_06945806 | 94 (97) |
| *orf36* | 41152 | 41505 | 117/366 | Transposase IS116/IS110/IS902 family protein | Transposase (*Finegoldia magna* ATCC 53516) | ZP_06945806 | 99 (99) |
| *orf37* | 41987 | 43318 | 446 | Relaxase | Relaxase (*Ureaplasma urealyticum* serovar 9 str. ATCC 33175) | ZP_03079581 | 96 (99) |
| *orf38* | 43320 | 43676 | 118 | - | Hypothetical protein Apre_0683 (*Anaerococcus prevotii* DSM 20548) | YP_003152443 | 98 (100) |
| *orf39* | 43855 | 44190 | 111 | - | Conserved domain protein (*Ruminococcus albus* 8) | ZP_06719337 | 33 (49) |
| *orf40* | 44183 | 44416 | 77 | - | No homology |  |  |
| *orf41* | 44523 | 45005 | 160 | - | Conserved hypothetical protein (*Finegoldia magna* BVS033A4) | ZP_07321893 | 93 (96) |
| *orf42* | 45014 | 45562 | 182 | - | Hypothetical protein MGAS10750_Spy1713 (*Streptococcus pyogenes* MGAS10750*)* | YP_603207 | 98 (98) |
| *orf43* | 45571 | 45972 | 133 | Transcriptional regulator | Transcriptional regulator, Cro/CI family (*Streptococcus pyogenes* MGAS10750) | YP_603208 | 99 (100) |
| *orf44* | 46122 | 46682 | 186 | - | Hypothetical protein MGAS10750_Spy1715 (*Streptococcus pyogenes* MGAS10750) | YP_603209 | 98 (100) |
| *orf45* | 46952 | 47362 | 136 | - | Sigma-70, region 4 family (*Ureaplasma urealyticum* serovar 9 str. ATCC 33175) | ZP_03079532 | 99 (99) |
| *orf46* | 47839 | 48060 | 73 | - | Conserved hypothetical protein (*Finegoldia magna* BVS033A4) | ZP_07321660 | 97 (97) |
| *orf47* | 48161 | 49831 | 556 | Site-specific recombinase | Site-specific recombinase family protein (*Ureaplasma urealyticum* serovar 9 str. ATCC 33175) | ZP_03079551 | 99 (99) |
| *orf48* | 49831 | 51501 | 556 | Site-specific recombinase | Site-specific recombinase (*Streptococcus pyogenes* MGAS10750) | YP_603212 | 98 (99) |
| *orf49* | 51494 | 52987 | 497 | Site-specific recombinase | Site-specific recombinase (*Streptococcus pyogenes* MGAS10750) | YP_603213 | 97 (99) |
