# Supplementary material for: Complete genome sequence of a serotype 11A, ST62 Streptococcus pneumoniae invasive isolate
Source: BMC Microbiol. 2011 Feb 1;11:25. doi: 10.1186/1471-2180-11-25 (PMC3055811; doi:10.1186/1471-2180-11-25)
Supplement: Additional file 3 — Figure S3. Schematic representation of Tn1806 of S. pneumoniae AP200, in comparison with the predicted genetic element of F. magna ATCC29328. This figure describes in detail the regions of similarity between the two genetic elements. [file 1471-2180-11-25-S3.PPT]

## Slide 1
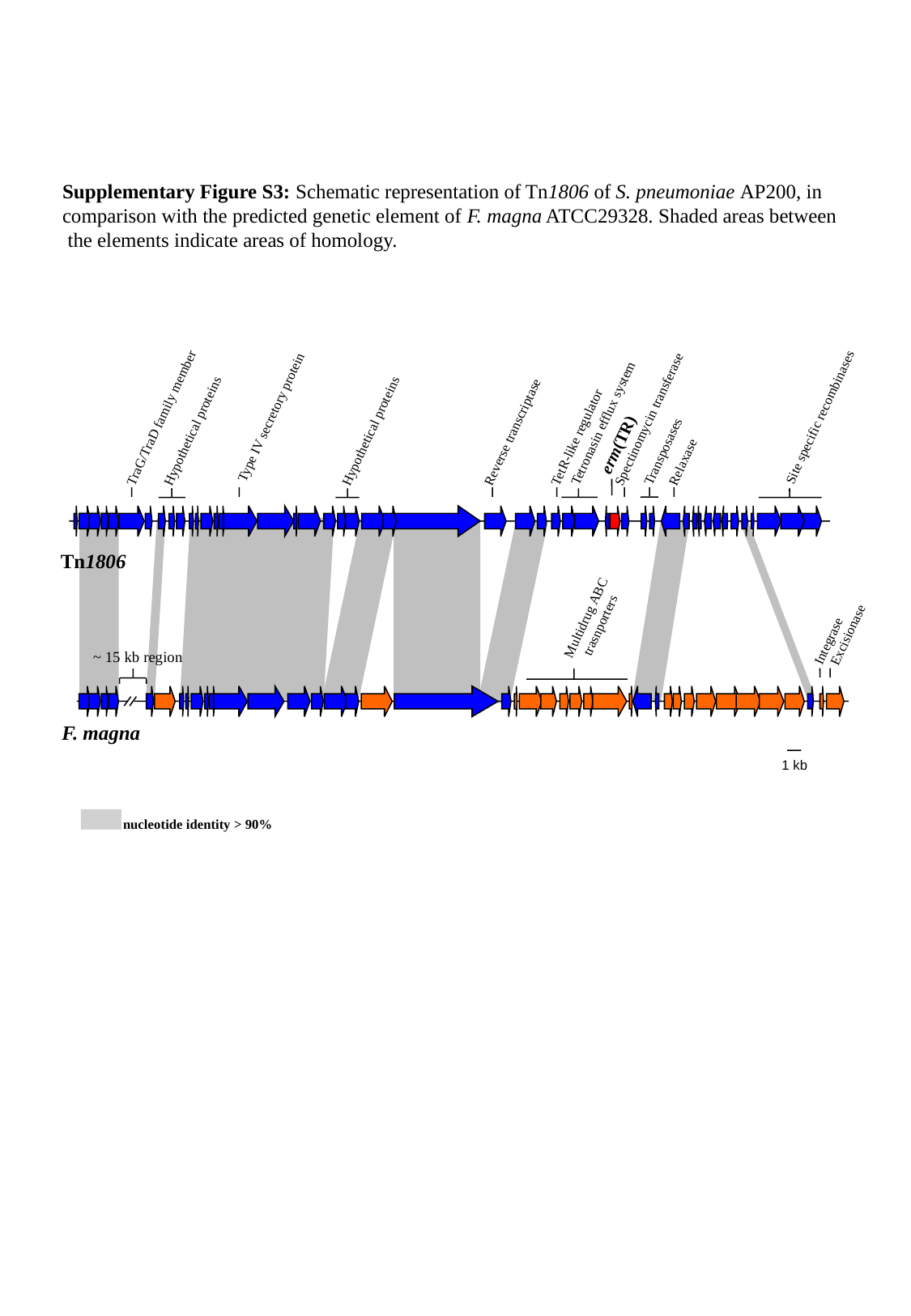

Supplementary Figure S3: Schematic representation of Tn1806 of S. pneumoniae AP200, in
comparison with the predicted genetic element of F. magna ATCC29328. Shaded areas between
 the elements indicate areas of homology.
Type IV secretory protein
Site specific recombinases
TraG/TraD family member
Spectinomycin transferase
Tetronasin efflux system
Hypothetical proteins
Hypothetical proteins
Reverse transcriptase
TetR-like regulator
erm(TR)
Transposases
Relaxase
Tn1806
Multidrug ABC
 trasnporters
Excisionase
Integrase
~ 15 kb region
F. magna
1 kb
nucleotide identity > 90%
