# Supplementary material for: Complete genome sequence of a serotype 11A, ST62 Streptococcus pneumoniae invasive isolate
Source: BMC Microbiol. 2011 Feb 1;11:25. doi: 10.1186/1471-2180-11-25 (PMC3055811; doi:10.1186/1471-2180-11-25)
Supplement: Additional file 4 — Table S4. Comparative analysis of the genes from ϕSpn_200 with proteins included in the databases. This table summarizes the homologies of the ORFs of ϕSpn_200 with proteins included in current databases. [file 1471-2180-11-25-S4.DOC]

**Supplementary Table S4.** Comparative analysis of the genes from φSpn_200 with proteins included in the databases. The Start and the Stop codons of the ORFs are referred to CP002121.

| **ORF** | **Start** | **Stop** | **Size**  **(amino acid)** | **Putative function** | **BLASTP best match** | **Accession no.** | **% Amino acid**  **identity**  **(% similarity)** |
| --- | --- | --- | --- | --- | --- | --- | --- |
| *orf1* | 23468 | 24616 | 382 | Integrase | integrase  (*S. pneumoniae* CDC1873-00) | ZP_02708689.1 | 100 (100) |
| *orf2* | 24787 | 25587 | 266 |  | hypothetical protein  (*S. pneumoniae* Hungary19A-6) | YP_001693452.1 | 100 (100) |
| *orf3* | 25714 | 26469 | 251 | Transcriptional repressor | phage transcriptional repressor (*S. pneumoniae* CDC1873-00) | ZP_02708687.1 | 100 (100) |
| *orf4* | 26643 | 26849 | 68 | Transcriptional regulator Cro/CI family | conserved domain protein  (*S. pneumoniae* Hungary19A-6) | YP_001693454.1 | 100 (100) |
| *orf5* | 26871 | 27032 | 53 |  | hypothetical protein  (*S. pneumoniae* CGSSp14BS69) | ZP_01828891.1 | 81 (94) |
| *orf6* | 27066 | 27194 | 42 |  | hypothetical protein  (*S. pneumoniae* Hungary19A-6);  gp13 (*S. mitis* phage SM1) | YP_001693456.1  NP_862852.1 | 100 (100)  88 (92) |
| *orf7* | 27157 | 27834 | 225 |  | gp14  (*S. pneumoniae* Hungary19A-6);  gp14 (*S. mitis* phage SM1) | YP_001693457.1  NP_862853.1 | 99 (100)  93 (97) |
| *orf8* | 27889 | 28602 | 237 | Prophage antirepressor | gp15  (*S. pneumoniae* Hungary19A-6);  gp15 (*S. mitis* phage SM1) | YP_001693458.1  NP_862854.1 | 100 (100)  87 (94) |
| *orf9* | 28615 | 28872 | 85 |  | Phage protein  (*S. pneumoniae* CDC1873-00) | ZP_02964472.1 | 100 (100) |
| *orf10* | 28958 | 29278 | 106 |  | hypothetical protein  (*S. pneumoniae* Hungary19A-6) | YP_001693460.1 | 100 (100) |
| *orf11* | 29294 | 29587 | 97 |  | gp18  (*S. pneumoniae* CDC1873-00) | ZP_02708680.1 | 100 (100) |
| *orf12* | 29571 | 30407 | 278 |  | gp19  (*S. pneumoniae* CDC1873-00)  hypothetical protein  (*S. pneumoniae* Hungary19A-6)  gp19 (*S. mitis* phage SM1) | ZP_02708679.2  YP_001693462.1  NP_862858.1 | 100 (100)  98 (98)  49 (65) |
| *orf13* | 30395 | 30553 | 52 |  | hypothetical protein  (*S. pneumoniae* Hungary19A-6) | YP_001693463.1 | 100 (100) |
| *orf14* | 30547 | 31317 | 256 | DNA replication protein | hypothetical protein  (*S. pneumoniae* Hungary19A-6) | YP_001693464.1 | 100 (100) |
| *orf15* | 31332 | 31526 | 64 |  | hypothetical protein  (*S. pneumoniae* CDC1873-00) | ZP_02708676.1 | 100 (100) |
| *orf16* | 31526 | 31753 | 75 |  | hypothetical protein  (*S. pneumoniae* CGSSp11BS70) | ZP_01826406.1 | 100 (100) |
| *orf17* | 31746 | 31850 | 34 |  | hypothetical protein  (*S. pneumoniae* CDC1873-00) | ZP_02708675.1 | 100 (100) |
| *orf18* | 31938 | 32147 | 69 |  | hypothetical protein  (*S. pneumoniae* CGSSp14BS69) | ZP_01828905.1 | 100 (100) |
| *orf19* | 32119 | 32436 | 105 |  | hypothetical protein MM1p22  (*S. pneumoniae* phage MM1) | NP_150152.1 | 94 (98) |
| *orf20* | 32438 | 33133 | 231 |  | hypothetical protein MM1p23  (*S. pneumoniae* phage MM1) | NP_150153.1 | 69 (81) |
| *orf21* | 33371 | 33724 | 117 | Immunity repressor protein | Cro/CI family transcriptional regulator prophage LambdaSa2  (*S. agalactiae* 2603V/R) | NP_688851.1 | 51 (71) |
| *orf22* | 33706 | 34170 | 154 |  | hypothetical protein  (*S. pneumoniae* CDC1873-00);  hypothetical protein, prophage LambdaSa2 (*S. agalactiae* 2603V/R) | ZP_02708668.1  NP_688850.1 | 100 (100)  80 (88) |
| *orf23* | 34279 | 34821 | 180 | Site- specific recombinase | Phage integrase family protein  prophage LambdaSa2  (*S. agalactiae* 2603V/R) | NP_688849.1 | 100 (100) |
| *orf24* | 35027 | 35254 | 75 |  | no significant homology found |  |  |
| *orf25* | 35251 | 35568 | 105 | HNH endonuclease | HNH endonuclease family protein  (*S. pneumoniae* Hungary19A-6);  HNH endonuclease family protein  prophage LambdaSa2  (*S. agalactiae* 2603V/R) | YP_001693471.1  NP_688847.1 | 100 (100)  89 (96) |
| *orf26* | 35705 | 36190 | 161 |  | hypothetical protein  (*S. pneumoniae* CGSSp14BS69);  hypothetical protein, prophage LambdaSa2 (*S. agalactiae* 2603V/R) | ZP_01828912.1  NP_688846.1 | 99 (100)  98 (99) |
| *orf27* | 36183 | 37895 | 570 | Terminase | Phage terminase, large subunit  (*S. pneumoniae* Hungary19A-6);  Phage terminase, large subunit,  prophage LambdaSa2  (*S. agalactiae* 2603V/R) | YP_001693473.1  NP_688845.1 | 100 (100)  94 (98) |
| *orf28* | 37904 | 39046 | 380 |  | hypothetical protein  (*S. pneumoniae* CDC1873-00);  hypothetical protein, prophage LambdaSa2 (*S. agalactiae* 2603V/R) | ZP_02708663.1  NP_688844.1 | 100 (100)  86 (94) |
| *orf29* | 39093 | 39635 | 180 | Prohead maturation protease | Phage prohead protease  (*S. pneumoniae* CDC1873-00);  Protease, prophage LambdaSa2  (*S. agalactiae* 2603V/R) | ZP_02708662.1  NP_688843.1 | 100 (100)  92 (96) |
| *orf30* | 39650 | 40903 | 417 | Minor structural protein | hypothetical protein  (*S. pneumoniae* Hungary19A-6);  hypothetical protein, prophage LambdaSa2 (*S. agalactiae* 2603V/R) | YP_001693476.1  NP_688842.1 | 100 (100)  74 (87) |
| *orf31* | 40929 | 41264 | 111 |  | hypothetical protein  (*S. pneumoniae* CDC1873-00);  hypothetical protein, prophage LambdaSa2 (*S. agalactiae* 2603V/R) | ZP_02708660.1  NP_688841.1 | 100 (100)  73 (85) |
| *orf32* | 41261 | 41566 | 101 |  | Putative phage head-tail adaptor  (*S. pneumoniae* CDC1873-00);  hypothetical protein, prophage LambdaSa2 (*S. agalactiae* 2603V/R) | ZP_02708659.1  NP_688840.1 | 100 (100)  88 (95) |
| *orf33* | 41566 | 41913 | 115 |  | hypothetical protein  (*S. pneumoniae* CDC1873-00);  hypothetical protein, prophage LambdaSa2 (*S. agalactiae* 2603V/R) | ZP_02708658.1  NP_688839.1 | 100 (100)  87 (95) |
| *orf34* | 41900 | 42244 | 114 |  | hypothetical protein  (*S. pneumoniae* Hungary19A-6);  hypothetical protein, prophage LambdaSa2 (*S. agalactiae* 2603V/R) | YP_001693480.1  NP_688838.1 | 100 (100)  77 (87) |
| *orf35* | 42258 | 42926 | 222 | Major tail protein | Phage major tail protein, Phi13 family (*S. pneumoniae* Hungary19A-6);  hypothetical protein, prophage LambdaSa2 (*S. agalactiae* 2603V/R) | YP_001693481.1  NP_688837.1 | 100 (100)  87 (93) |
| *orf36* | 42928 | 43404 | 158 |  | hypothetical protein  (*S. pneumoniae* Hungary19A-6);  hypothetical protein, prophage LambdaSa2 (*S. agalactiae* 2603V/R) | YP_001693482.1  NP_688836.1 | 100 (100)  94 (96) |
| *orf37* | 43443 | 43571 | 42 |  | hypothetical protein  (*S. pneumoniae* CDC1873-00);  hypothetical protein, prophage LambdaSa2 (*S. agalactiae* 2603V/R) | ZP_02708654.1  NP_688835.1 | 100 (100)  90 (97) |
| *orf38* | 43591 | 46329 | 912 | Tail tape measure protein | Tail tape measure family protein  (*S. pneumoniae* Hungary19A-6);  hypothetical protein, prophage LambdaSa2 (*S. agalactiae* 2603V/R) | YP_001693484.1  NP_688834.1 | 99 (100)  85 (93) |
| *orf39* | 46326 | 47048 | 240 | Tail component protein | hypothetical protein  (*S. pneumoniae* Hungary19A-6);  hypothetical protein, prophage LambdaSa2 (*S. agalactiae* 2603V/R) | YP_001693485.1  NP_688833.1 | 99 (100)  76 (88) |
| *orf40* | 47049 | 56156 | 3035 | Minor structural protein | PblB (*S. pneumoniae* Hungary19A-6) | YP_001693486.1 | 97 (98) |
| *orf41* | 56250 | 56453 | 67 |  | hypothetical protein  (*S. pneumoniae* P1031);  hypothetical protein  (*S. pneumoniae* Hungary19A-6) | YP_002737313.1  YP_001693487.1 | 98 (98)  91 (95) |
| *orf42* | 56456 | 56806 | 116 |  | hypothetical protein  (*S. pneumoniae* Hungary19A-6);  hypothetical protein, prophage LambdaSa2 (*S. agalactiae* 2603V/R) | YP_001693488.1  NP_688833.1 | 99 (100)  76 (85) |
| *orf43* | 56816 | 57232 | 138 | Holin 1 | Putative holin 1  (*S. pneumoniae* Hungary19A-6) | YP_001693489.1 | 100 (100) |
| *orf44* | 57236 | 57568 | 110 | Holin | Phage holin, LL-H family (*S. pneumoniae* P1031);  Phage holin (*S. pneumoniae* Hungary19A-6) | YP_002737317.1  YP_001693490.1 | 99 (100)  92 (98) |
| *orf45* | 57572 | 58528 | 318 | Autolysin | Autolysin (*S. pneumoniae* CGSSp11BS70);  Autolysin (*S. pneumoniae* Hungary19A-6) | ZP_01824138.1  YP_001693491.1 | 98 (99)  98 (98) |
| *orf46* | 58666 | 58845 | 59 |  | hypothetical protein  (*S. pneumoniae* CDC1873-00) | ZP_02708644.1 | 100 (100) |
| *orf47* | 58987 | 59142 | 51 |  | hypothetical protein (*S. pneumoniae* CGSSp11BS70) | ZP_01824141.1 | 98 (100) |
