# Supplementary material for: Complete genome sequence of a serotype 11A, ST62 Streptococcus pneumoniae invasive isolate
Source: BMC Microbiol. 2011 Feb 1;11:25. doi: 10.1186/1471-2180-11-25 (PMC3055811; doi:10.1186/1471-2180-11-25)
Supplement: Additional file 5 — Figure S5. Phage plaque assay using the S. pneumoniae indicator strain Rx1. This figure shows the Rx1 lawn lysis due to ϕSpn_200 activity. [file 1471-2180-11-25-S5.PPT]

## Slide 1
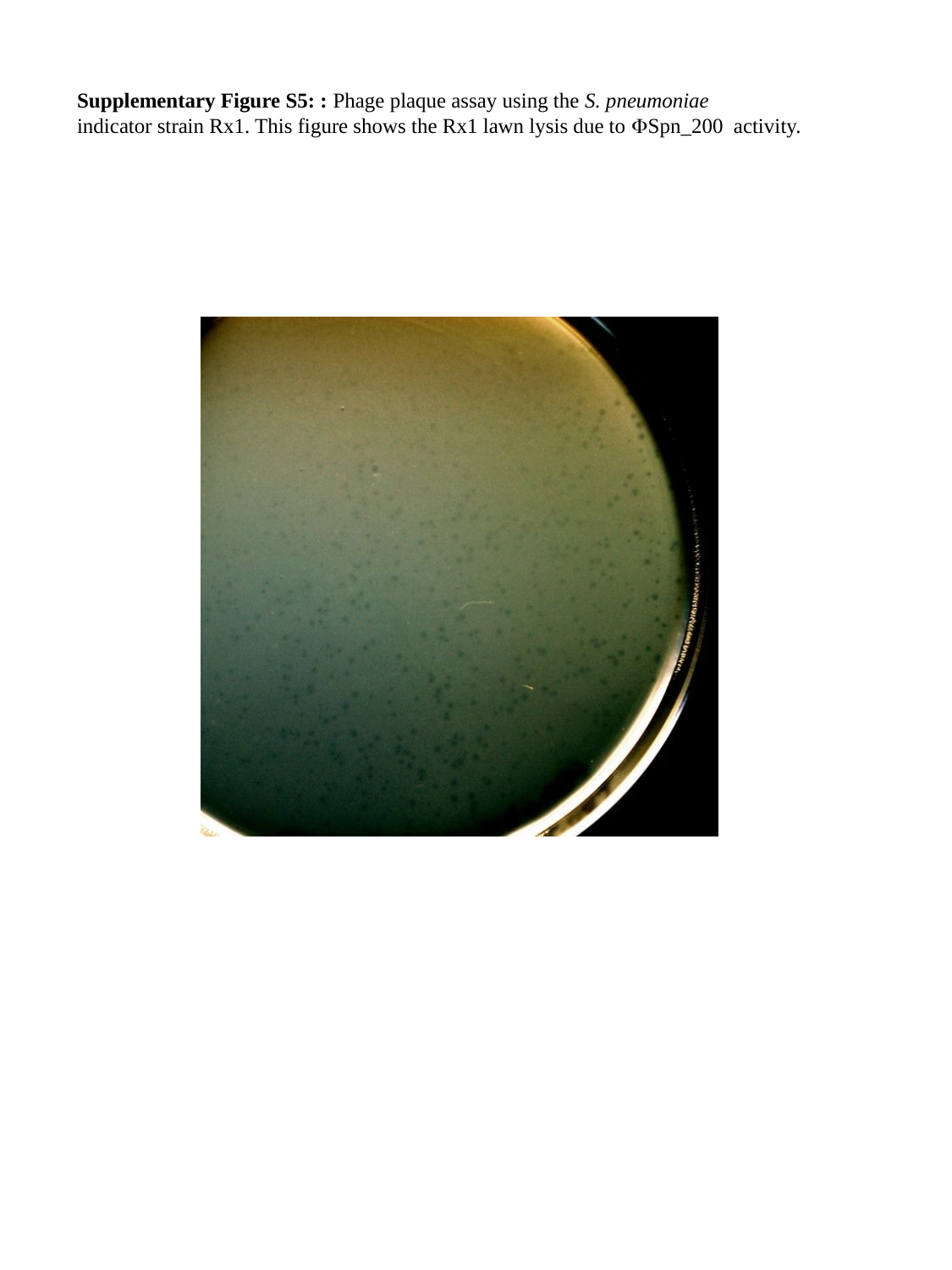

Supplementary Figure S5: : Phage plaque assay using the S. pneumoniae
indicator strain Rx1. This figure shows the Rx1 lawn lysis due to Spn_200 activity.
